# *Ehrlichia chaffeensis* Infection of Sika Deer, Japan

**DOI:** 10.3201/eid1512.081667

**Published:** 2009-12

**Authors:** Makoto Kawahara, Tomoko Tajima, Harumi Torii, Mitsutaka Yabutani, Joji Ishii, Makiko Harasawa, Emiko Isogai, Yasuko Rikihisa

**Affiliations:** Nagoya City Public Health Research Institute, Nagoya, Japan (M. Kawahara, M. Yabutani, J. Ishii); Osaka Prefecture University, Sakai, Japan (T. Tajima); Nara University of Education, Nara, Japan (H. Torii); Kyoto University, Kyoto, Japan (M. Harasawa); Health Sciences University of Hokkaido, Tobetsu, Japan (E. Isogai); The Ohio State University, Columbus, Ohio, USA (Y. Rikihisa)

**Keywords:** Ehrlichia chaffeensis, sika deer, GroEL, 16S rRNA, bacteria, Japan, dispatch

## Abstract

To determine whether *Ehrlichia chaffeensis* exists in Japan, we used PCR to examine blood from sika deer in Nara, Japan. Of 117 deer, 36 (31%) were infected with *E. chaffeensis*. The *E. chaffeensis* 16S rRNA base and GroEL amino acid sequences from Japan were most closely related to those of *E. chaffeensis* Arkansas.

Human infection with *Ehrlichia chaffeensis* causes human monocytic ehrlichiosis (HME), an influenza-like illness. Severity of the disease varies from mild to severe and can even cause death (*1*). HME cases have been reported primarily in the southeastern and south–central regions of the United States (*1*).

The organism, *E. chaffeensis,* until recently has been reported only in the United States; however, numerous reports now indicate that *Ehrlichiae* spp. closely related or identical to *E. chaffeensis* exist throughout the world (*1*,*2*). In the United States, *E. chaffeensis* has been most frequently identified in the lone star tick (*Amblyomma americanum*) (*3*). *E. chaffeensis* DNA has also been detected in *A. testudinarium* and *Haemaphysalis yeni* ticks from southern People’s Republic of China (*4*), in *H. longicornis* ticks from South Korea (*5*), and in *A. parvum* ticks in Argentina (*6*). Other than *A. americanum*, the role of these tick species as *E. chaffeensis* vectors has not been investigated. The white-tailed deer (*Odocoileus virginianus*) is the only vertebrate species currently recognized as a complete and sufficient host for maintaining the transmission cycle of *E. chaffeensis* (*3*,*7*). To look for molecular evidence of *E. chaffeensis* in sika deer (*Cervus nippon*) in Japan, we examined blood specimens by using PCR amplification of the 16S rRNA and *groEL* genes.

## The Study

In Nara, Japan, to prevent injuries to park visitors, the Foundation for the Protection of Sika Deer in Nara Park captured 97 pregnant female sika deer in April 2005 and 20 male sika deer with antlers in November 2005. Blood specimens were collected from all 117 deer, and buffy coat fractions were stored at –80°C for further analysis. Genomic DNA was extracted from the buffy coat fractions by using a QIAamp tissue kit (QIAGEN, Valencia, CA, USA). Nested PCR amplification of genomic DNA was performed by using primer pairs designed to amplify the *E. chaffeensis* 16S ribosomal RNA (rRNA) gene and the *E. chaffeensis* *groEL* gene (Table).

**Table:** Detection of 16S rRNA gene and *groEL* gene of *Ehrlichia chaffeensisin* in sika deer, Japan

Target gene	1st PCR or nested PCR	Primer ID	Product size, bp	Sequence of primers (5′ → 3′)	No. positive
16S rRNA	1st	NS16SCH1F	1195	ACGGACAATTGCTTATAGCCTT	7
		NS16SCH1R		ACAACTTTTATGGATTAGCTAAAT	
	Nested	NS16SCH2F	443	GGGCACGTAGGTGGACTAG	36
		NS16SCH2R		CCTGTTAGGAGGGATACGAC	
*groEL* gene	1st	NSgroCH1F	849	GTTGTAACTGGTGAACAACTC	4
		NSgroCH1R		CTTTTCTTCTATCACCAAACCC	
	Nested	NSgroCH2F	469	GTTCGTATTTTGGAAGATGCTG	35
		NSgroCH2R		ACTGTGATAACTCCATCCTTAC	

Of the 117 specimens, 36 (31%) yielded *E. chaffeensis* 16S rRNA amplification products and 35 (30%) yielded *E. chaffeensis groEL* amplification products. Of 36 16S rRNA–positive specimens, 33 were positive for *groEL* (92% concordance rate). Of 35 *groEL*-positive specimens, 33 were positive for 16S rRNA (94% concordance rate). The *E. chaffeensis* sequences were conserved in that all sequences obtained from sika deer in Nara were nearly identical to those of a representative strain that we named NS101 and submitted to GenBank (accession no. AB454074). The sequence of the 16S rRNA gene from *E. chaffeensis* NS101 was most closely related (99.6% identity; 5 bases of 1,333 bp that can be aligned for comparison differed) to that of 5 human isolates: Arkansas (GenBank accession no. M73222) (*8*), Sapulpa (U60476) (*9*), 91HE17 (U23503) (*10*), St. Vincent (U86665) (*11*), and Jax (U86664) (*11*); the next closest sequence was from an *E. chaffeensis* isolate from *A. testudinarium* ticks in China (GenBank accession no. AF147752) (99.2% identity; 10 bases of 1,333 bp that can be aligned for comparison differed).

**Figure 1:**
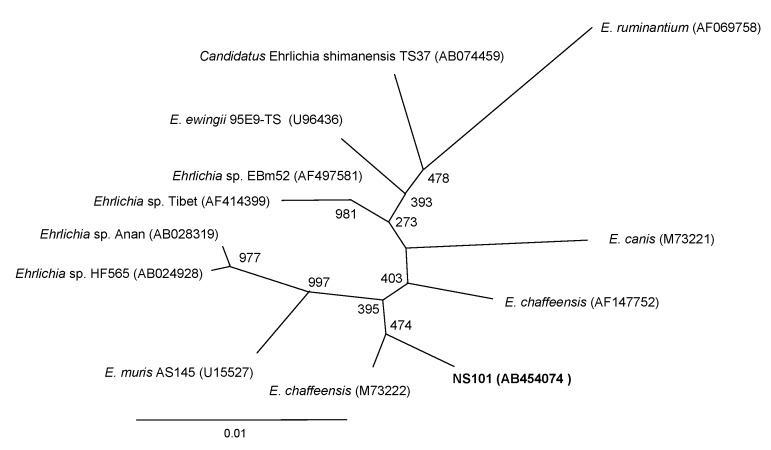
Phylogenetic relationship between *Ehrlichia chaffeensis* NS101 (in **boldface**) and other *Ehrlichia* spp. 16S rRNA gene sequences. GenBank accession numbers are shown in parentheses. Numbers above internal nodes indicate the number of bootstrap replicates of 1,000 that supported the branch. Scale bar indicates percent sequence divergence.

When *E. chaffeensis* NS101 was compared with *Ehrlichia* spp. previously identified in Japan, the sequence of the 16S rRNA gene (a 1,328-bp segment that can be aligned for comparison) of *E. chaffeensis* NS101 was 98.9%, 98.9%, and 98.6% identical to that of *E. muris* AS145 strain, *Ehrlichia* sp. HF565 (the HF strain, *Ixodes ovatus* Ehrlichia) and *Candidatus* Ehrlichia shimanensis TS37, respectively. Phylogenic analysis concurred with the observation that *E. chaffeensis* from Nara sika deer has the highest identity to the *E. chaffeensis* human isolates from the United States (Figure 1).

Although longer 16S rRNA gene sequences are desirable for strain comparison, the sequence of the 16S rRNA gene of *E*. *chaffeensis* from *H. longicornis* ticks in Korea (GenBank accession no. AY350424, 390 bp) and from *A. parvum* ticks in Argentina (GenBank accession no. EU826516, 470 bp) were identical to a corresponding, but incomplete, segment (358 bp and 402 bp, respectively) of the 16S rRNA gene of *E. chaffeensis* NS101 and Arkansas strains. An *Ehrlichia* sp. was detected in blood samples from marsh deer (*Blastocerus dichotomus*) captured near the Parana River in southeast Brazil in 1998 (*12*). However, the 16S rRNA sequence from these marsh deer (GenBank accession no. DQ345720, 383 bp) shares 98.7% identity to a corresponding, but incomplete, segment (381 bp) of the *E. chaffeensis* NS101 and Arkansas strains. Because the sequences of the corresponding segments of *Ehrlichia* sp. HF565 and ‘*Candidatus* Ehrlichia shimanensis’ TS37 both had 99.0% identity to that of *E. chaffeensis* Arkansas strain, higher than the 98.7%, the *Ehrlichia* sp. in marsh deer from Brazil might not be *E. chaffeensis.*

**Figure 2:**
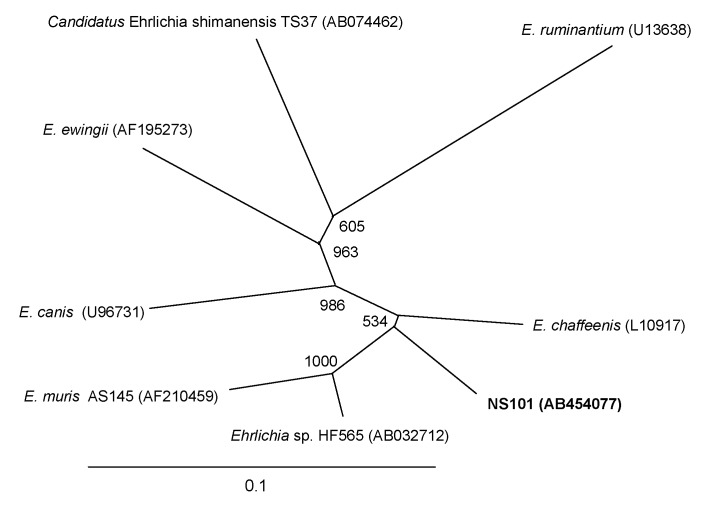
Phylogenetic relationship between the *Ehrlichia chaffeensis* NS101 *groEL* sequence (1,208 bp) (in **boldface**) and other *Ehrlichia* spp. *groEL* sequences. GenBank accession numbers are shown in parentheses. Numbers above internal nodes indicate the number of bootstrap replicates of 1,000 that supported the branch. Scale bar indicates percent sequence divergence.

Among the *E. chaffeensis groEL* sequences available in current databases, only that from *E. chaffeensis* Arkansas has >1,000 bp for reliable comparison. The *groEL* DNA sequence (1,208 bp that can be aligned) of the NS101 strain (GenBank accession no. AB454077, 1,311 bp) was most closely related to that of *E. chaffeensis* Arkansas (GenBank accession no. L10917), followed by the *Ehrlichia* sp. HF565 strain (GenBank accession no. AB032712), and the *E. muris* AS145 strain (GenBank accession no. AF210459) (Figure 2). The deduced *E. chaffeensis* NS101 GroEL amino acid sequence (402 residues) was most closely related (99.5% identity) to that of *E. chaffeensis* Arkansas, followed by the *Ehrlichia* sp. HF565 strain (99.2% identity) and *E. muris* AS145 strain (99.0%).

## Conclusions

On the basis of the long 16S rRNA and the *groEL* DNA sequences, our study demonstrates the presence of *E. chaffeensis* in sika deer from Nara, Japan. The genetic similarity of *E. chaffeensis* in the sika deer in Japan to strains isolated from HME patients in the United States raises the possibility that HME may exist in Japan. Of 1,803 serum samples collected from persons in metropolitan Tokyo from 1991 through 1995, when *E. muris* was used as antigen, 20 were seropositive (*13*). Because *E. chaffeensis* and *E. muris* antigens are highly cross-reactive (*14*), some of these persons might have been infected with *E. chaffeensis*. Of the 10 tick species found on sika deer (*15*), the primary tick species found on sika deer in Nara is *H. longicornis,* which is known to bite humans in Japan and to be infected with *E. chaffeensis* in South Korea (*5*). Because sika deer are abundant and increasing throughout Japan (*16*), this finding highlights need to survey sika deer and humans in Japan for *E. chaffeensis* infection.
